# Prevalence and associated factors of antibiotic self-medication and home storage among antibiotic users: a cross-sectional study in Vietnam

**DOI:** 10.1186/s12889-025-23202-4

**Published:** 2025-05-26

**Authors:** Dung Anh Doan, An Duc Nguyen, Giang Ba Le, Thuy Thi Xuan Nguyen, Phuong Lan Nguyen, Dai Xuan Dinh

**Affiliations:** 1 Faculty of Pharmacy, Phenikaa University, Hanoi, Vietnam; 2 Faculty of Pharmaceutical Management and Economics, Hanoi University of Pharmacy, 13-15 Le Thanh Tong, Hoan Kiem District, Hanoi City, Vietnam; 3 Faculty of Public Health, Pham Ngoc Thach University of Medicine, Ho Chi Minh, Vietnam; 4 Department of Pharmacy, Da Nang University of Medical Technology and Pharmacy, Da Nang, Vietnam; 5 Drug Administration of Vietnam, Ministry of Health, Hanoi, Vietnam

**Keywords:** Antibiotic, Associated factor, Home storage, Self-medication, User, Vietnam

## Abstract

**Background:**

Antibiotic self-medication and home storage are two common behaviors in the community that can lead to inappropriate or unnecessary use. This study investigated the prevalence and factors associated with these two behaviors among antibiotic users in Vietnam.

**Methods:**

In this cross-sectional study, 997 participants from six cities/provinces were selected using a convenience sampling method and directly interviewed from November 2023 to March 2024. Antibiotic home storage was assessed at the time of interviewing, while self-medication was assessed for the year right before this time. Factors associated with antibiotic self-medication and home storage were identified via multivariate logistic regression models and the Bayesian Model Averaging method.

**Results:**

About 35.8% of the participants self-medicated with antibiotics. Among these 357 individuals, the main rationales behind this behavior were mild diseases (46.8%), time-saving (37.8%), and easy access to antibiotics from community pharmacies (33.6%). Antibiotics for self-medication were obtained mainly via community pharmacies without prescriptions (71.7%). Sore throat (45.7%), cough/common cold (42.6%), fever (37.8%), and runny nose/stuffy (31.9%) were the top four diseases/symptoms behind antibiotic self-medication. Besides, 27.3% stored antibiotics at home. Most were leftovers from previous treatments (69.1%) or deliberately reserved (33.1%). Factors associated with antibiotic self-medication included the participants’ knowledge about antibiotics (aOR = 0.96, 95%CI: 0.94–0.98), the number of people living with the participant (aOR = 1.16, 95%CI: 1.04–1.30), purchasing antibiotics without a prescription (aOR = 5.09, 95%CI: 3.78–6.85), and storing antibiotics at home (aOR = 3.52, 95%CI: 2.55–4.86). Region (north: aOR = 4.72, 95%CI: 3.15–7.08), area (urban: aOR = 0.60, 95%CI: 0.40–0.89), sharing antibiotics with others (aOR = 1.96, 95%CI: 1.38–2.79), having leftover antibiotics (aOR = 3.35, 95%CI: 2.34–4.79), and self-medicating with antibiotics in the past year (aOR = 2.97, 95%CI: 2.15–4.10) were significantly associated with home storage of antibiotics.

**Conclusions:**

Antibiotic self-medication and home storage were prevalent among Vietnamese people. Health education programs should be implemented to raise public awareness about the potential risks of these two behaviors, thereby contributing to lower inappropriate antibiotic use.

**Supplementary Information:**

The online version contains supplementary material available at 10.1186/s12889-025-23202-4.

## Introduction

In recent years, antimicrobial resistance has been a global public health concern. In 2019, an estimated 4.95 million deaths were associated with bacterial antimicrobial resistance, including 1.27 million deaths attributable to this issue [1]. By 2050, this global issue is predicted to be attributed to one hundred trillion dollars in societal and financial costs [2]. Antimicrobials include four main subgroups: antibiotics, antivirals, antiparasitics, and antifungals [3]. Among them, antibiotics are the medicine group that should be paid attention to by virtue of overuse and misuse in the community. Inappropriate antibiotic use may result in worsening disease conditions, increasing treatment costs, and developing side effects, drug interactions, and recurrent infections [4]. This behavior is also a primary driver of antibiotic resistance [5]. Besides misuse and overuse, the lack of new antibiotic development and manufacturing in the pharmaceutical industry has also contributed to the antibiotic resistance crisis [6].

Antibiotic self-medication and home storage are two common behaviors in the community. As per the World Health Organization, self-medication involves “the use of medicinal products by the consumer to treat self-recognized disorders or symptoms, or the intermittent or continued use of a medication prescribed by a physician for chronic or recurring diseases or symptoms” [7]. It also includes the use of family members’ medication [7]. There has been an alarmingly high prevalence of antibiotic self-medication in many countries. A systematic review and meta-analysis showed that the overall prevalence of antibacterial self-medication was 38.8% in developing countries. Many antibiotics (such as ampicillin, tetracycline, metronidazole, ceftriaxone, and cotrimoxazole) were commonly used to treat symptoms of viral infections [8]. This figure varied among countries but was substantially high in low- and middle-income countries [9–11]. Besides self-medication, storing antibiotics at home is not recommended, as this behavior can lead to inappropriate antibiotic use. A study showed that antibiotic home storage was found among 48.1% of Chinese parents, and this behavior was significantly associated with self-medication with antibiotics for their children [12]. In Mecha, Ethiopia, more than a fifth of the households stored antimicrobials in their home with a condition similar to household materials [13]. In common, medicines should be stored in a cool and dry place with a temperature of below 25^o^C. This issue is of particular concern in tropical countries like Vietnam, as storing antibiotics in unsuitable conditions can increase the risk of medicine deterioration and expiration, thereby leading to medicine toxicity [14].

Although antibiotic self-medication and home storage are rampant in many countries, and numerous studies have been conducted to assess these behaviors, data in Vietnam are scant, especially in recent years. During the 1990s, several studies were conducted to assess antibiotic self-medication and non-prescription antibiotic dispensing practices in Vietnam. Some issues were reported, such as an increase in self-medication practice when a person stored medicines in the house and mistaken beliefs about antibiotics among citizens [15–17]. Another study showed that many people even used antibiotics for various symptoms and conditions often without requiring these medicines [18]. Recently, some studies have been conducted to investigate the knowledge, attitudes, and practices about antibiotics in Vietnam [18, 19]. However, little specific information involving antibiotic self-medication and home storage was reported. This study was carried out to investigate the prevalence and associated factors of antibiotic self-medication and home storage among antibiotic users in Vietnam during 2023–2024.

## Methods

### Study design, setting, and period

This cross-sectional study was conducted in Vietnam from November 2023 to March 2024. Vietnam can be divided into three parts. In each part, two cities/provinces were purposely selected to collect data (north: Hanoi and Haiduong, central: Nghean and Danang, and south: Hochiminh and Vinhlong).

### Population and sample size calculation

Participants’ inclusion criteria were Vietnamese citizens, at least 18 years old, and using antibiotics at least once during the year right before the time they were interviewed. We collect data from antibiotic users because this is a part of our project to assess the practices of Vietnamese people involving antibiotic use. We did not include non-users, as they had no relevant practices to be surveyed. Healthcare staff (such as doctors, nurses, pharmacists, and allied health professionals) and medical students were excluded. The sample size was computed using the formula: n = Z_α/2_^2^.p.(1-p)/d^2^. With α = 0.01, Z_α/2_=2.575, *p* = 0.5, and d = 0.05, the minimum sample size was 663 people.

### Development and validation of the questionnaire

Our questionnaire was developed based on relevant documents and published scientific articles [6, 13, 20–31] and included three main parts: (1) participants’ demographic characteristics, (2) the knowledge and health beliefs/attitudes toward antibiotics, and (3) some practices involving antibiotics (mainly focusing on antibiotic self-medication and home storage).

Participants’ demographic characteristics included their year of birth, sex, region, area, level of education, marital status, the number of people living with the participant, working status, participant’s monthly income, family’s monthly income, whether or not living with a person working in the medical field, the frequency of seeking health information, and source of information about antibiotics and health problems. Two scales used to measure participants’ knowledge and health beliefs/attitudes toward antibiotics can be seen in Table S1. The former included fifteen questions about antibiotics and antibiotic resistance, while the latter consisted of eleven questions about perceived benefits, threats, and self-efficacy of behaviors involving inappropriate antibiotic use. Cronbach’s alpha was 0.73 (knowledge) and 0.76 (health belief/attitudes), indicating acceptable internal consistency.

Two primary outcomes of this study were the prevalence of antibiotic self-medication and home storage. Regarding self-medication with antibiotics, participants were asked, “*How many times did you use antibiotics without seeing a doctor in the past year?*”. Then, for people self-medicating with antibiotics, the investigators continued to collect their data about antibiotics’ names, conditions treated with antibiotics, reasons for self-medication, their selection of antibiotics, and antibiotic usage during self-medication. The participants were also asked whether or not they had the intention to self-medicate with antibiotics in the future. The second primary outcome was antibiotic storage at home, measured by asking, “*Are you storing any kind of antibiotics at home? (not for current use)*”. People with a “Yes” answer were also asked about the names of antibiotics stored at their homes, where they had the antibiotics, reasons for storage, type of antibiotics, places to store antibiotics, and whether or not stored antibiotics were expired. Besides these behaviors, the participants were also asked whether or not they shared (gave/received) antibiotics with other people, purchased antibiotics without a prescription, took antibiotics for disease prevention, and had leftover antibiotics (not completing a treatment course) during the year before the time of interviewing.

### Sampling technique, procedures, and data collection

The participants were recruited in each city/province using a convenience sampling method. Trained investigators were lecturers/researchers and university students. The participants were approached in their houses or public places (such as parks or malls). After an introduction to the study’s name and objectives, eligible people were invited to participate in this survey. They were interviewed face-to-face for approximately fifteen minutes. The questionnaire was designed as a Google Form link. Ipads and similar electronic gadgets were used to collect data.

### Statistical analysis

Data were analyzed using R software version 4.4.0. Numeric variables were described via mean and standard deviation (SD). Categorical variables were reported via numbers and percentages. Binary logistic regression models were employed to determine factors associated with antibiotic self-medication and home storage among Vietnamese people. The Bayesian Model Averaging method was employed to select independent variables in the multivariate models. To assess the goodness-of-fit of these models, the research team employed the Hosmer-Lemeshow test and calculated the area under the curve (AUC) and Nagelkerke’s R-squared value. A *p*-value < 0.05 was considered statistically significant.

## Results

### Participants’ demographic characteristics

A total of 1,424 Vietnamese people were approached. Eighty-one did not agree to participate in this research or worked/studied in the medical field (response rate: 94.3%). Among 1,343 people agreeing to participate in this study, 346 did not use antibiotics in the past year. Finally, 997 individuals were included in the analysis. Among them, 56.6% were male. Their average age was 38.5 ± 14.6 years old. Nearly half had an education level of high school, while a quarter graduated from a university. Nearly two-thirds came from rural areas (63.1%) and got married (62.4%). There was at least one medical worker in the family of about 26.3% of the participants. Roughly 67.9% were working at the point of interviewing. Nearly two-thirds (61.9%) had a monthly income/allowance/pension of five million Vietnam dongs or higher. The total monthly income of their families was mostly five million Vietnam dongs or higher (93.8%). The participants usually used the Internet (70.3%), medical personnel (59.8%), and mass media (41.8%) to gather information about health (Table 1).

**Table 1:** Main characteristics of the participants

No	Characteristics (*N* = 997 participants)		*n*	%
1	Sex	Male	564	56.6
		Female	433	43.4
6	Age (years old)	< 20	31	3.1
		20–29	283	28.4
		30–39	210	21.1
		40–49	213	21.4
		> 50	260	26.1
2	Region	North	358	35.9
		Central	251	25.2
		South	388	38.9
3	Area	Rural	629	63.1
		Urban	368	36.9
4	Level of education	Secondary school or lower	166	16.6
		High school	466	46.7
		College/Intermediate	117	11.7
		University/post-university	248	24.9
5	Marital status	Single/Widow	375	37.6
		Married	622	62.4
6	Having a family member who is a medical worker	Yes	262	26.3
		No	735	73,7
7	Occupation	Not working/Studying/Retired	320	32.1
		Working	677	67.9
8	Participant’s average monthly income, allowance, pension (mVNDs)	< 5	380	38.1
		5 to < 10	281	28.2
		10 to < 15	159	15.9
		15 or higher	177	17.8
9	Family’s average monthly income (mVNDs)	< 5	62	6.2
		5 to < 10	125	12.5
		10 to < 15	186	18.7
		15 or higher	624	62.6
10	Seeking health information	Never/rarely	194	19.5
		Sometimes	554	55.6
		Usually	249	25.0
11	Source of information about antibiotics and health problems	The Internet (social networks, online papers…)	701	70.3
		Medical personnel (doctors, nurses…)	596	59.8
		Mass media (television, radio…)	417	41.8
		Friends, family members, relatives	390	39.1
		Books and paper documents	314	31.5
		Website of reliable organizations (MOH, WHO…)	218	21.9
		Scientific articles (PubMed, Google Scholar…)	121	12.1
		Relevant courses	40	4.0

**Fig. 1:**
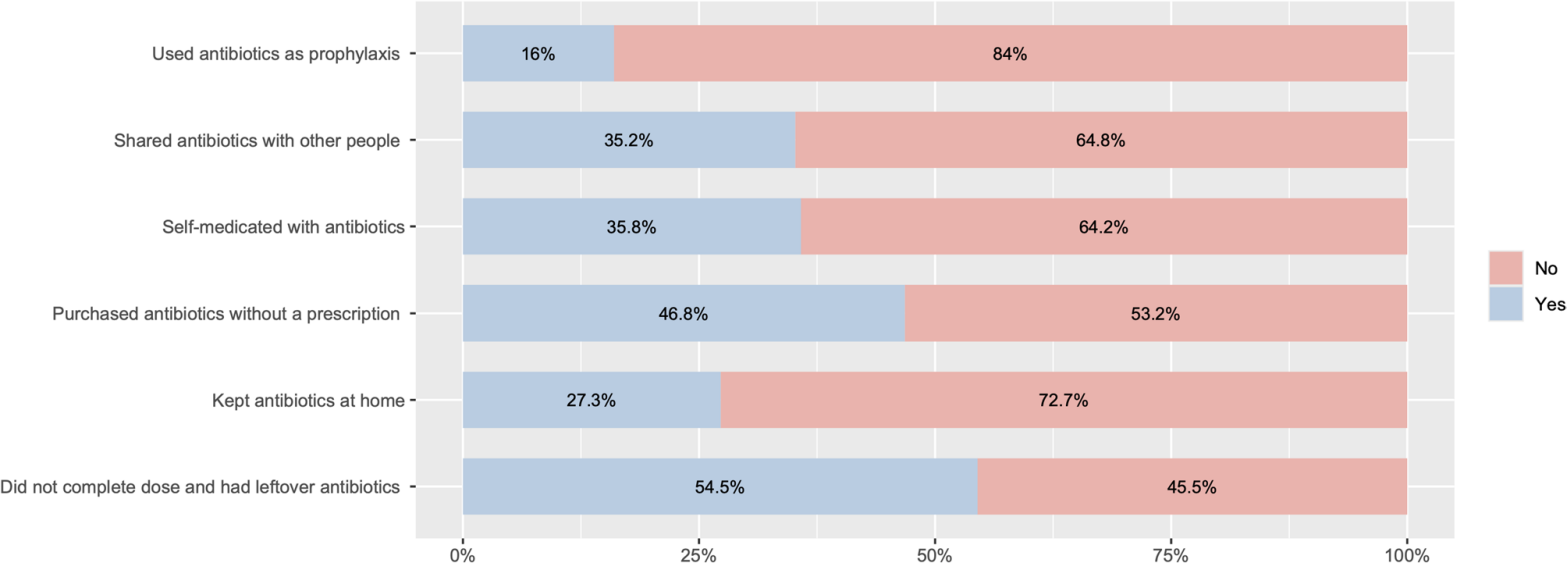
Some behaviors involving antibiotic use among Vietnamese people in the past year

In the past year, 35.2% of participants shared (gave/received) antibiotics with other people. Nearly half (46.8%) purchased antibiotics without having prescriptions. Approximately 16.0% took this medication to prevent disease. In addition, 54.5% had leftover antibiotics. Participants’ average knowledge score about antibiotics was 14.290 ± 5.930 (possible scores: from 0 to 30). Their average attitude score was 40.682 ± 6.337 (possible scores: from 11 to 55) (Fig. 1).

### Self-medication with antibiotics

In the past year, 357 individuals self-medicated with antibiotics at least once (35.8%, 95% confidence interval (95%CI): 32.8-38.8%) (Fig. 1). The top five reasons for this inappropriate behavior were mild diseases (46.8%), saving time (37.8%), easy access to antibiotics from community pharmacies (33.6%), complicated medical examination process (23.8%), and having a home stock of antibiotics (22.4%). The antibiotics for self-medication were obtained mainly via community pharmacies without prescriptions (71.7%). The participants selected antibiotics for self-medication based on community pharmacists’ consultancy (57.1%), previous prescriptions (26.1%), their family members, friends, relatives (25.5%), and personal knowledge/experience (23.5%). Sore throat (45.7%), cough/common cold (42.6%), fever (37.8%), and runny nose/stuffy (31.9%) were the top four diseases/symptoms behind antibiotic self-medication. Amoxicillin/acid clavulanic, ciprofloxacin, and cotrimoxazole were the three kinds of antibiotics most commonly used for self-medication (34.5%, 14.3%, and 13.7%, respectively) (Table 2, Table S2).

**Table 2:** Self-medication with antibiotics among antibiotic users in Vietnam (main results)

No	Characteristics (*N* = 357 people self-medicating with antibiotics)		*n*	%
1	Reasons for self-medication with antibiotics	Low severity of disease	167	46.8
		No time to see a doctor (time-saving)	135	37.8
		Easy access to antibiotics from community pharmacies	120	33.6
2	Where did you usually obtain antibiotics from for self-medication?	Community pharmacies	256	71.7
		Home storage	69	19.3
		Leftovers	54	15.1
3	Selected antibiotics based on…	Community pharmacists’ consultancy	204	57.1
		Previous prescriptions	93	26.1
		Family members, friends, relatives	91	25.5
		Personal knowledge/experience	84	23.5
4	What did you consider when selecting antibiotics?	Indications for use	217	60.8
		Brand names/manufacturers	152	42.6
		Price	141	39.5
		Side effects, adverse reactions, allergies	122	34.2
5	Diseases, symptoms	Sore throat	163	45.7
		Cough/common cold	152	42.6
		Fever	135	37.8
		Runny nose/stuffy	114	31.9
6	Type of antibiotics	Amoxicillin/acid clavulanic	123	34.5
		Ciprofloxacin	51	14.3
		Cotrimoxazole	49	13.7
7	Source of drug information	Community pharmacists	221	61.9
		The patient information leaflet	185	51.8
		Personal knowledge/experience	71	19.9
8	Duration of antibiotic self-medication (days)	1–3	131	36.7
		4–7	151	42.3
		> 7	41	11.5
9	When did you normally stop taking antibiotics?	After symptoms disappeared	207	58.0
		At the completion of the course	150	42.0
10	Changed the dosage of antibiotics during the course of self-treatment		100	28.0
11	Switched antibiotics during the course of self-treatment		80	22.4
12	Took the same antibiotics with different names at the same time during the course of self-treatment		29	8.1
13	Had any adverse reactions when self-medicating with antibiotics		75	21.0
14	Had intention to self-medication in the future		226	63.3

During the course of self-treatment, 100 participants (28.0%) changed the dosage of antibiotics because their conditions improved (59.0%) or worsened (41.0%). Eighty people (22.4%) switched antibiotics because the former antibiotic did not work (77.5%) and/or they wanted to reduce adverse reactions (32.5%). About 8.1% took the same antibiotics with different names at the same time. Roughly 21.0% thought they had at least one adverse reaction when self-medicating with antibiotics. Approximately 58.0% stopped taking antibiotics after symptoms disappeared, while 42.0% completed the treatment course. Nearly two-thirds (63.3%) had intention to self-medicate with antibiotics in the future (Table 2, Table S2).

### Home storage of antibiotics

Among 997 participants, 272 (27.3%, 95%CI: 24.5-30.1%) stored antibiotics at home for use at a later time (Fig. 1). Two-thirds (69.1%) stored antibiotics at home because they had leftovers from missed doses, while a third (33.1%) deliberately reserved these medicines for future use when needed. The participants usually stored tablets (48.5%) and capsules (37.1%). Nearly three-quarters (72.8%) stored antibiotics in boxes, drawers, or medicine cabinets, while more than a quarter put antibiotics on tables or shelves (27.2%). Amoxicillin/acid clavulanic (36.0%), cephalexin (12.1%), tobramycin (8.5%), and ciprofloxacin (7.4%) were the four common types of antibiotics stored at home (Table 3).

**Table 3:** Home storage of antibiotics among antibiotic users in Vietnam

No	Characteristics (*N* = 272 people storing antibiotics at their home)		*n*	%
1	From where you had antibiotics to store	Leftovers from missed doses	188	69.1
		Community pharmacies (without a prescription)	90	33.1
		From the Internet	24	8.8
		Others (from family members/friends…)	23	8.5
2	Type of stored antibiotics	Tablet	132	48.5
		Capsule	101	37.1
		Solution/suspension	36	13.2
		Powder	36	13.2
		Cream/gel/oilment	21	7.7
3	Place for storage	In boxes, drawers, or medicine cabinets	198	72.8
		On tables or shelves	74	27.2
		In refrigerators	8	2.9
4	Name of stored antibiotics	Amoxicillin/acid clavulanic	98	36.0
		Cephalexin	33	12.1
		Tobramycin	23	8.5
		Ciprofloxacin	20	7.4
		Cotrimoxazole	18	6.6
		Metronidazole/spiramicin	18	6.6
		Azithromycin	16	5.9
		Ampicillin	15	5.5
		Cefixime	10	3.7
		Cefuroxime	9	3.3
		Tetracyclin	8	2.9
		Levofloxacin	8	2.9
		Neomycin	6	2.2
		Penicillin	6	2.2
		Others (Bacitracin, Ofloxacin…)	43	15.8
5	Expiration date of stored antibiotics at home (at the time of data collection)	Not expired	212	77.9
		Expired	8	2.9
		Do not know	52	19.1

### Factors associated with antibiotic self-medication and home storage among Vietnamese people

Self-medication with antibiotics was significantly associated with participants’ knowledge about antibiotics, the number of people living with the participant, purchasing antibiotics without a prescription, and home storage of antibiotics. In particular, individuals purchasing antibiotics without a prescription (adjusted odds ratio (aOR) = 5.09, 95%CI: 3.78–6.85, *p* < 0.001) and storing antibiotics at home (aOR = 3.52, 95%CI: 2.55–4.86, *p* < 0.001) were more likely to self-medicate with antibiotics when compared with those who did not. Regarding the knowledge about antibiotics, each additional increase of one score was associated with a 4% decrease in the odds of antibiotic self-medication (aOR = 0.96, 95%CI: 0.94–0.98, *p* = 0.001). By contrast, this odds increased by 16% for each increase of one person living with the participant (aOR = 1.16, 95%CI: 1.04–1.30, *p* = 0.009) (Table 4). As per univariate analyses, self-medication with antibiotics was also more prevalent among individuals sharing antibiotics with other people and having leftover antibiotics in the past year (*p* < 0.001) (Table S3).

**Table 4:** Factors associated with antibiotic self-medication and home storage among antibiotic users in Vietnam

Independent variable	Self-medication with antibiotics		Home storage of antibiotics	
	aOR (95% CI)	*p*-value	aOR (95% CI)	*p*-value
1. Region: North (ref: Central and South)			4.72 (3.15–7.08)	< 0.001
2. Area: Urban (ref: Rural)			0.60 (0.40–0.89)	0.011
3. The number of people living with the participant	1.16 (1.04–1.30)	0.009		
4. Knowledge about antibiotics	0.96 (0.94–0.98)	0.001		
5. Shared antibiotics with other people in the past year (ref: No)			1.96 (1.38–2.79)	< 0.001
6. Purchased antibiotics without a prescription (ref: No)	5.09 (3.78–6.85)	< 0.001		
7. Had leftover antibiotics in the past year (ref: No)			3.35 (2.34–4.79)	< 0.001
8. Stored antibiotics at home (ref: No)	3.52 (2.55–4.86)	< 0.001		
9. Self-medicated with antibiotics in the past year (ref: No)			2.97 (2.15–4.10)	< 0.001
Hosmer-Lemeshow test	X-squared = 5.6461, df = 8, *p*-value = 0.687		X-squared = 5.0059, df = 8, *p*-value = 0.757	
Area under the curve (AUC)	0.768 (0.737–0.798)		0.772 (0.740–0.803)	
Nagelkerke’s R-squared	0.270		0.264	

ref: reference, aOR: adjusted odds ratio, 95% CI: 95% confidence interval, mVND: million Vietnam dongs (1mVND = 41.66US$)

Factors associated with antibiotic home storage included region, area, sharing antibiotics with others, having leftover antibiotics, and self-medicating with antibiotics in the past year. The participants living in the north were 4.72 times (95%CI: 3.15–7.08, *p* < 0.001) more likely to store antibiotics at home than those living in other regions. This behavior was less prevalent among those living in urban areas (aOR = 0.60, 95%CI: 0.40–0.89, *p* = 0.011) than those from rural areas. Those not completing their doses (having leftover antibiotics) (aOR = 3.35, 95%CI: 2.34–4.79, *p* < 0.001), self-medicating with antibiotics (aOR = 2.97, 95%CI: 2.15–4.10, *p* < 0.001), and sharing antibiotics with other people (aOR = 1.96, 95%CI: 1.38–2.79, *p* < 0.001) were also associated with a higher likelihood of storing antibiotics at home (Table 4). Furthermore, older people and the married were more likely to store antibiotics at home than other groups (*p* < 0.001) (Table S3).

## Discussion

This study investigated the prevalence and factors associated with antibiotic self-medication and home storage among 997 Vietnamese people using antibiotics in the past year. The results showed that more than a third self-medicated with antibiotics at least once. The main rationales behind this behavior were mild diseases, time-saving, and easy access to antibiotics from community pharmacies. Antibiotics for self-medication were obtained mainly via community pharmacies without prescriptions. Besides, more than a quarter stored antibiotics at home. Most of them were leftovers from missed doses or deliberately reserved. Amoxicillin/acid clavulanic was the antibiotic most commonly used for self-medication and home storage. Factors significantly associated with the former included the participants’ knowledge about antibiotics, the number of people living with the participant, purchasing antibiotics without a prescription, and storing antibiotics at home. Region, area, sharing antibiotics with others, having leftover antibiotics, and self-medicating with antibiotics were significantly associated with the latter.

In this study, 35.8% (95%CI: 32.8-38.8%) of the participants self-medicated with antibiotics at least once. In many other countries, the prevalence of antibiotic self-medication was far higher than our result (for example, Pakistan (2019): 59.6% (95%CI: 57.5-61.7%) [32] and Colombia (2022): 46% (95%CI: 42.5-49.5%) [33]). The high prevalence of people self-medicating with antibiotics in these studies can be attributed to the availability and easy access to antibiotics in pharmacies. In Vietnam, recently, the government and the Ministry of Health have strived to put a curb on trading antibiotics without prescriptions. Meanwhile, in Pakistan, it was very common for people to buy antibiotics without proper prescriptions since the regulations were not strict enough to hinder this activity [32]. Besides, the public’s knowledge and beliefs about antibiotics can be another reason [33]. Previous studies showed that the general public’s knowledge of antibiotics/antibiotic use was extremely low [10, 22, 32]. Lacking the necessary knowledge and self-medicating with antibiotics can give rise to incorrect choice, dosage, and duration of therapy, treatment failures, safety issues (such as masking or worsening conditions, adverse reactions, and drug interactions), and developing antibiotic resistance [4, 34]. However, some studies reported lower figures (for example, Malaysia (2019): 15.1% (95%CI: 11.9-18.3%) [35] and Sri Lanka (2016–2017): 10.8% (95%CI: 8.9-12.7%) [36]). The differences among studies may spring from the differences in locations, studying time, sampling methods, and the definition of self-medication. In this study, we only calculated the percentage of antibiotic self-medication among antibiotic users. This criterion was not included in the two above studies. Besides, we investigated antibiotic self-medication for a year before the time the participants were interviewed. In the study from Sri Lanka, the authors assessed this behavior for only the last time the participants took an antibiotic in the past three months [36]. This can contribute to lowering the figure from this country.

Vietnamese people’s main reasons for antibiotic self-medication were mild diseases, time-saving, easy access to antibiotics from community pharmacies, complicated medical examination process, and home storage. A systematic review also indicated that in low- and middle-income countries, patient-related determinants (such as perceived low severity of disease, intention to save time, and having home stock of antibiotics) and health system-related determinants (such as high cost of consulting physicians and easy access to antibiotics from pharmacies) were rationales behind this behavior [34]. It is noteworthy that Vietnamese people took antibiotics to self-treat sore throat, cough, common cold, runny nose/stuffy, and fever. They were also the major complaints that resulted in antibiotic self-medication among many people in Eritrea, Ethiopia, Sri Lanka, and Pakistan [10, 11, 28, 29]. In many cases, these health conditions/symptoms are originally viral and not bacterial infections. Therefore, using antibiotics is unnecessary [4]. In Vietnam, antibiotics are prescription-only medicines. Although the Ministry of Health and relevant organizations have strived to curb the trade in antibiotics without prescriptions, people still have easily purchased these medicines in community pharmacies. Other studies also highlighted medicine outlets in the community as familiar sources of antibiotics for self-medication [35, 37]. As a result, community pharmacists can play a crucial role in counseling and educating the general public to help them understand relevant regulations and rational antibiotic use. Heavy sentences should be imposed for pharmacists violating laws by selling antibiotics without prescriptions.

Home storage of antibiotics was found among 272 Vietnamese people (27.3%, 95%CI: 24.5-30.1%). This behavior can give rise to other issues, such as inappropriate utilization and disposal. Home storage of antibiotics was also prevalent among many people in Australia (46.9%, 95%CI: 44.6-49.2%) [38] and Ethiopia (antimicrobials: 21.2%, 95%CI: 18.5-23.9%) [13]. In Vietnam, most stored antibiotics were from leftovers and/or deliberately reserved (directly purchased over the counter). Tablets and capsules were the two common dosage forms stored at home, and only a few expired, similar to the results of a study in Lebanon for leftover antibiotics [39]. In Ethiopia, capsules and tablets constituted 87.5% of stored antimicrobials. However, at the time of data collection, 28.8% of stored antimicrobials were expired [13]. Besides, similar to our finding, amoxicillin alone or in combination with acid clavulanic was also the antibiotic most commonly used for self-medication and/or stored at home among the general population in Eritrea, Ethiopia, Pakistan, Colombia, and Malaysia [10, 11, 13, 28, 33, 35]. Affordable costs, various dosage forms, safety profile, and high availability in community pharmacies could explain their frequent use and storage in many countries, including Vietnam [40, 41].

Storing antibiotics at home was associated with a greater chance of self-medication. This relationship was also demonstrated in some previous studies. Among Vietnamese people, self-medication was significantly associated with home storage (aOR = 3.52, 95%CI: 2.55–4.86, *p* < 0.001), consistent with the result of a study from the United States (storage and intended use: OR = 5.3, 95%CI: 2.7–10.5) [42]. Besides, family members could be a crucial factor associated with these inappropriate behaviors. Households with larger family sizes could be more likely to contract contagious diseases. When the number of people in a family increases, the possibility of self-medication, home storage, and sharing medicines among members may also increase [13]. In many families, ailments are not only personal but also family problems. In many cultures, women are the people who bear responsibility for taking care of other members. Health education campaigns should focus on these influential people who can be the bridge to promote the rational use of medicines to other family members [4].

Another factor associated with self-medication with antibiotics was participants’ knowledge of antibiotics and antibacterial resistance. Vietnamese people with lower knowledge scores were more likely to self-medicate with antibiotics (aOR = 0.96, 95%CI: 0.94–0.98, *p* = 0.001), in line with the results of studies in Eritrea (inadequate knowledge: aOR = 2.13, 95%CI: 1.12–4.05) [10] and Pakistan (having higher knowledge levels: aOR = 0.82, 95%CI:0.67–0.99, *p* = 0.042) [32]. A systematic review also reported that people with greater knowledge in low- and middle-income countries were less likely to self-medicate with antibiotics [34]. Regarding home storage of antibiotics, this behavior was also more prevalent among older people, consistent with the data of Mecha, Ethiopia [13]. Coming down with many diseases and having difficulties in movement may be potential reasons for storing medicines at home among the elderly. Besides the above factors, these two antibiotic-related issues were more prevalent among those purchasing antibiotics without prescriptions, not completing the treatment course/having leftovers, and sharing antibiotics with other people. As a result, there is a connection among inappropriate antibiotic use behaviors. Education campaigns need to be comprehensively developed to hinder antibiotic misuse and guarantee that the general public can have adequate knowledge about rational medicine use. The government should have strict disciplinary regulation policies to control antibiotic trade and use in the community. In this study, we only focused on investigating two issues involving antibiotics. Other studies are needed to obtain a complete picture of antibiotic misuse among the general population in Vietnam.

To the best of our knowledge, this is the first study investigating the prevalence and factors associated with antibiotic self-medication and home storage among Vietnamese people after the COVID-19 pandemic. Some strengths included having a large sample size, directly interviewing the participants, and using the Bayesian Model Averaging method to select independent variables in multivariate models. The similarity between the sample and population in some characteristics (including age, sex, and area) can help increase the generalization and extrapolation of findings. However, this study has several limitations. First, this is a cross-sectional study, so the causal inference between dependent and independent variables cannot be established. Second, using a convenience sampling method to recruit the participants may affect some independent variables (such as a high proportion of people living with a medical worker) and lower the generalization of the findings. Besides, we also only collected data on antibiotic users during the past year. As a result, the findings cannot be representative of the general population. Third, asking people about antibiotic use during the past year can lead to recall bias. Recruiting participants in public places can also affect some results (such as whether or not stored antibiotics were expired). Lastly, in some cases, a person cannot know all kinds of antibiotics because there are numerous substances categorized as antibiotics and a multitude of brands in the market. This issue can lower the percentage of antibiotic self-medication and home storage among antibiotic users in our study.

## Conclusions

Antibiotic self-medication and home storage were prevalent among Vietnamese people. Factors significantly associated with self-medication included their knowledge about antibiotics, the number of people living with the participant, purchasing antibiotics without a prescription, and storing antibiotics at home. Home storage of antibiotics was significantly associated with region, area, sharing antibiotics with other people, having leftover antibiotics, and self-medication. Our results supported the need for health education and multifaceted intervention programs targeting the general population, especially antibiotic users, to reduce unnecessary/inappropriate practices, such as self-medication and home storage. Healthcare professionals and policymakers can make a contribution to raising the public’s knowledge about appropriate antibiotic use and put a curb on trading these medications without prescriptions in community pharmacies.

## Electronic supplementary material

Below is the link to the electronic supplementary material.

**Supplementary Material 1**: **Additional file 1**

## Data Availability

The datasets used and/or analyzed during the current study are available from the corresponding author upon reasonable request.

## Abbreviations

aOR: Adjusted odds ratio
AUC: The area under the curve
CI: Confidence interval
COVID-19: Coronavirus disease 2019
OR: Odds ratio
SD: Standard deviation
