# Supplementary material for: Prevalence and associated factors of antibiotic self-medication and home storage among antibiotic users: a cross-sectional study in Vietnam
Source: BMC Public Health. 2025 May 26;25:1940. doi: 10.1186/s12889-025-23202-4 (PMC12105128; doi:10.1186/s12889-025-23202-4)
Supplement: Supplementary file 1 — Supplementary Material 1: Additional file 1 [file 12889_2025_23202_MOESM1_ESM.pdf]

Table S1. Knowledge and attitude items and scoring

A. Knowledge items	True	Probably true	False	Probably false	Do not know
1. Antibiotics are effective for treating both bacteria and viral infections.	0	0	2	1	0
2. Antibiotics have pain-relieving and fever-reducing effects similar to paracetamol.	0	0	2	1	0
3. If a person suffers from a cough, runny nose, and a sore throat, he or she will recover faster if he or she takes antibiotics as soon as possible.	0	0	2	1	0
4. Antibiotics can be taken with various types of drinks (such as milk and fruit juice) to limit their bitter taste.	0	0	2	1	0
5. Higher doses lead to faster treatment and recovery.	0	0	2	1	0
6. Expensive antibiotics are more effective and have fewer side effects (than cheaper ones).	0	0	2	1	0
7. People should be stopped taking antibiotics when symptoms are relieved.	0	0	2	1	0
8. Antibiotics can cause side effects (such as diarrhea, allergic reactions, and even death).	2	1	0	0	0
9. An antibiotic will always be effective in treating the same infection in the future (Antibiotics maintain their effectiveness over time).	0	0	2	1	0
10. If bacteria become resistant to antibiotics, it will be difficult or impossible to treat the infections they cause.	2	1	0	0	0

11. There are bacteria infections resistant to all available antibiotics.	2	1	0	0	0
12. Antibiotic resistance is a serious problem in other countries but not in Vietnam.	0	0	2	1	0
13. Antibiotic resistance is only a problem for people who use antibiotics inappropriately.	0	0	2	1	0
14. Antibiotic-resistant bacteria can spread from animals or people to other people.	2	1	0	0	0
15. Using antibiotics for a duration shorter than that indicated or with low doses does not contribute to antibiotic resistance.	0	0	2	1	0
**B. Health beliefs/attitude items**	**Totally disagree**	**Disagree**	**Normal, neutral**	**Agree**	**Totally agree**
1. It is good to store antibiotics at home to use when needed.	5	4	3	2	1
2. It is okay to use antibiotics from friends, family members, and relatives if they are used to treat the same illnesses.	5	4	3	2	1
3. I can take antibiotics myself when I have minor illnesses or symptoms similar to previous illnesses without seeing a doctor.	5	4	3	2	1
4. Imported antibiotics are more effective than domestically produced antibiotics.	5	4	3	2	1
5. Antibiotics should be sold without a doctor's prescription.	5	4	3	2	1
6. I can look up how to use antibiotics myself without consulting a doctor or pharmacist.	5	4	3	2	1

7. If my doctor does not prescribe antibiotics when I think they are needed, I will see another doctor or buy them at a pharmacy without a prescription.	5	4	3	2	1
8. It is better to take antibiotics that may not be necessary than to wait and see if I get better without taking antibiotics.	5	4	3	2	1
9. There is nothing I can do to help prevent antibiotic resistance.	5	4	3	2	1
10. I think the use of antibiotics in animal husbandry needs to be strictly monitored.	1	2	3	4	5
11. Scientists can always produce new antibiotics, so we don't need to worry about antibiotic resistance.	5	4	3	2	1

Table S2. Self-medication with antibiotics among antibiotic users in Vietnam (n=357 people)

(full answers)

No	Self-medication with antibiotics		n	%
1	Reasons for self-medication with antibiotics *(multiple choices)*	Low severity of disease (The disease was not serious)	167	46.8
		No time/lack of time to see a doctor (time-saving)	135	37.8
		Easy access to antibiotics from community pharmacies	120	33.6
		Complicated medical examination process	85	23.8
		Having a home stock of antibiotics	80	22.4
		Having previous experience	74	20.7
		Cost saving (high cost of consulting doctors)	70	19.6
		Lack of trust/confidence in doctors	11	3.1
2	Where did you usually obtain antibiotics from for self-medication? *(multiple choices)*	Community pharmacies	256	71.7
		Home storage	69	19.3
		Leftovers	54	15.1
		Family members, friends, relatives	35	9.8
		Online purchase	18	5.0

No	Self-medication with antibiotics		n	%
3	Selected antibiotics based on...<br>*(multiple choices)*	Community pharmacists' consultancy	204	57.1
		Previous prescriptions	93	26.1
		Family members, friends, relatives	91	25.5
		Personal knowledge/experience	84	23.5
		Searching from the Internet	33	9.2
4	What did you consider when selecting antibiotics?<br>*(multiple choices)*	Indications for use	217	60.8
		Brand names/manufacturers	152	42.6
		Price	141	39.5
		Side effects, adverse reactions, allergies	122	34.2
5	Diseases, symptoms<br>*(multiple choices)*	Sore throat	163	45.7
		Cough/common cold	152	42.6
		Fever	135	37.8
		Runny nose/stuffy	114	31.9
		Pains/aches	54	15.1

No	Self-medication with antibiotics		n	%
		Wound infection	53	14.8
		Tonsillitis	52	14.6
		Diarrhea	37	10.4
		Others (scalds...)	27	7.6
6	Type of antibiotics *(multiple choices)*	Amoxicillin/acid clavulanic	123	34.5
		Ciprofloxacin	51	14.3
		Cotrimoxazole	49	13.7
		Ampicillin	36	10.1
		Cefixime	30	8.4
		Cephalexin	24	6.7
		Azithromycin	19	5.3
		Tetracyclin	18	5.0
		Cefuroxime	16	4.5
		Metronidazole/spiramicin	14	3.9

No	Self-medication with antibiotics		n	%
		Tobramycin	13	3.6
		Cefpodoxime	12	3.4
		Penicillin	12	3.4
		Others (Neomycin, Erythromycin...)	22	6.2
7	Source of drug information (how to use antibiotics) *(multiple choices)*	Community pharmacists	221	61.9
		The patient information leaflet	185	51.8
		Personal knowledge/experience	71	19.9
		Friends, family members, relatives	54	15.1
		The Internet	44	12.3
		Previous prescriptions	42	11.8
8	Duration of antibiotic self-medication (days) *(multiple choices)*	1 - 3	131	36.7
		4 - 7	151	42.3
		> 7	41	11.5
		Do not remember	39	10.9

No	Self-medication with antibiotics		n	%
9	Changed the dosage of antibiotics during the course of self-treatment	No	257	72.0
		Yes, because of:	100	28.0
		Improving conditions	*59*	*59.0*
		Worsening conditions	*41*	*41.0*
		Reducing adverse reactions	*18*	*18.0*
10	Switched antibiotics during the course of self-treatment	No	277	77.6
		Yes, because:	80	22.4
		The former antibiotic did not work	*62*	*77.5*
		To reduce adverse reactions	*26*	*32.5*
11	Took the same antibiotics with different names at the same time during the course of self-treatment	Yes	29	8.1
		No	328	91.9
12	Had any adverse reactions when self-medicating with antibiotics	No	282	79.0
		Yes, and I did...	75	21.0
		Stopped taking antibiotics	*38*	*50.7*

No	Self-medication with antibiotics		n	%
		Switched to another antibiotic	*26*	*34.7*
		Consulted a doctor or pharmacist	*39*	*52.0*
		Consulted family members/relatives/friends	*10*	*13.3*
		Continued to use	*5*	*6.7*
13	When did you normally stop taking antibiotics?	After symptoms disappeared	207	58.0
		At the completion of the course	150	42.0
14	Had intention to self-medication in the future	Yes	226	63.3
		No	131	36.7

Table S3. Factors associated with antibiotic self-medication and home storage among antibiotic users in Vietnam (univariate analyses)

Independent variable	Self-medication with antibiotics		Home storage of antibiotics	
	OR (95% CI)	p-value	OR (95% CI)	p-value
1. Sex: Male (ref: Female)	0.93 (0.72-1.21)	0.598	0.96 (0.73-1.27)	0.789
2. Age	1.01 (1.00-1.01)	0.218	1.02 (1.01-1.03)	<0.001
3. Region (ref: Central)				
North	0.61 (0.43-0.85)	0.003	2.57 (1.78-3.72)	<0.001
South	0.66 (0.48-0.92)	0.013	0.87 (0.58-1.29)	0.475
4. Area: Urban (ref: Rural)	0.83 (0.63-1.09)	0.181	0.43 (0.31-0.58)	<0.001
5. Level of education (ref: College/Intermediate)				
Secondary school or lower	0.91 (0.56-1.47)	0.690	0.93 (0.56-1.53)	0.771
High school	0.66 (0.43-0.99)	0.045	0.64 (0.41-0.99)	0.043
University or higher	0.72 (0.46-1.14)	0.159	0.64 (0.40-1.03)	0.069
6. Marital status: Single/widow (ref: Married)	0.87 (0.67-1.14)	0.321	0.58 (0.43-0.78)	<0.001
7. The number of people living with the participant	1.13 (1.03-1.25)	0.014	0.96 (0.87-1.07)	0.500
8. Living with a medical worker (ref: No)	0.98 (0.73-1.32)	0.903	0.73 (0.53-1.02)	0.064
9. Occupation: Working (ref: Not working/Studying/Retired)	1.21 (0.92-1.61)	0.175	1.31 (0.96-1.78)	0.086

Independent variable	Self-medication with antibiotics		Home storage of antibiotics	
	OR (95% CI)	p-value	OR (95% CI)	p-value
10. Participant's average monthly income/allowance (mVNDs) (ref: 10 to 15)				
<5	0.93 (0.64-1.37)	0.727	0.72 (0.47-1.09)	0.119
5 to 10	0.94 (0.63-1.40)	0.750	1.03 (0.68-1.58)	0.880
15 or higher	0.94 (0.60-1.46)	0.773	0.99 (0.62-1.59)	0.971
11. Family's average monthly income (mVNDs) (ref: 10 to <15)				
<5	0.89 (0.49-1.62)	0.705	1.70 (0.88-3.29)	0.112
5 to <10	0.98 (0.61-1.56)	0.919	1.20 (0.69-2.10)	0.515
15 or higher	0.86 (0.62-1.21)	0.396	1.82 (1.22-2.73)	0.003
12. Seeking health information (ref: often)				
Rarely/never	1.00 (0.72-1.41)	0.978	1.00 (0.69-1.46)	0.997
Usually	0.81 (0.59-1.11)	0.182	1.48 (1.07-2.05)	0.019
13. Source of information about antibiotics and health problems				
The Internet (social networks, online papers…)	1.02 (0.77-1.36)	0.886	0.86 (0.64-1.16)	0.331
Medical personnel (doctors, nurses…)	0.78 (0.60-1.02)	0.071	1.05 (0.79-1.40)	0.728
Mass media (television, radio…)	1.37 (1.06-1.78)	0.018	1.03 (0.77-1.36)	0.859

Independent variable	Self-medication with antibiotics		Home storage of antibiotics	
	OR (95% CI)	p-value	OR (95% CI)	p-value
Friends, family members, relatives	0.85 (0.65-1.11)	0.242	0.89 (0.67-1.19)	0.432
Books and paper documents	1.16 (0.88-1.54)	0.282	0.85 (0.63-1.16)	0.308
Websites of reliable organizations (MOH, WHO...)	0.69 (0.50-0.95)	0.025	0.90 (0.64-1.27)	0.550
Scientific articles (PubMed, Google Scholar...)	0.73 (0.48-1.11)	0.139	0.82 (0.53-1.28)	0.383
Relevant courses	0.86 (0.44-1.68)	0.656	1.30 (0.66-2.55)	0.451
14. Knowledge about antibiotics	0.96 (0.94-0.98)	<0.001	1.04 (1.02-1.07)	0.001
15. Beliefs/attitudes towards antibiotics	0.97 (0.95-0.99)	0.012	0.98 (0.96-1.00)	0.115
16. Shared antibiotics with other people in the past year (ref: No)	2.31 (1.76-3.02)	<0.001	1.76 (1.32-2.34)	<0.001
17. Purchased antibiotics without a prescription in the past year (ref: No)	5.37 (4.04-7.14)	<0.001	1.58 (1.20-2.09)	0.001
18. Used antibiotics as prophylaxis in the past year (ref: No)	1.45 (1.02-2.04)	0.036	0.80 (0.54-1.19)	0.274
19. Had leftover antibiotics in the past year (ref: No)	2.00 (1.53-2.61)	<0.001	2.44 (1.81-3.29)	<0.001
20. Stored antibiotics at home (at the time of data collection) (ref: No)	3.21 (2.41-4.29)	<0.001		
21. Self-medicated with antibiotics in the past year (ref: No)			3.21 (2.41-4.29)	<0.001
ref: reference, OR: odds ratio, 95%CI: 95% confidence interval, mVND: million Vietnam dongs (1mVND=41.66US$), WHO: World Health Organization, MOH: Ministry of Health.				
